# Strain Gradient Modulated Exciton Evolution and Emission in ZnO Fibers

**DOI:** 10.1038/srep40658

**Published:** 2017-01-13

**Authors:** Bin Wei, Yuan Ji, Raynald Gauvin, Ze Zhang, Jin Zou, Xiaodong Han

**Affiliations:** 1 Beijing Key Laboratory and Institute of Microstructure and Property of Advanced Materials, Beijing University of Technology, Beijing 100124, China; 2 Materials Engineering, McGill University, Montréal, Québec, H3A 0C5, Canada; 3 Department of Materials Science, Zhejiang University, Hangzhou 310027, China; 4 Materials Engineering and Centre for Microscopy and Microanalysis, The University of Queensland, St. Lucia, QLD 4072, Australia

## Abstract

One-dimensional semiconductor can undergo large deformation including stretching and bending. This homogeneous strain and strain gradient are an easy and effective way to tune the light emission properties and the performance of piezo-phototronic devices. Here, we report that with large strain gradients from 2.1–3.5% μm^−1^, free-exciton emission was intensified, and the free-exciton interaction (FXI) emission became a prominent FXI-band at the tensile side of the ZnO fiber. These led to an asymmetric variation in energy and intensity along the cross-section as well as a redshift of the total near-band-edge (NBE) emission. This evolution of the exciton emission was directly demonstrated using spatially resolved CL spectrometry combined with an *in situ* tensile-bending approach at liquid nitrogen temperature for individual fibers and nanowires. A distinctive mechanism of the evolution of exciton emission is proposed: the enhancement of the free-exciton-related emission is attributed to the aggregated free excitons and their interaction in the narrow bandgap in the presence of high bandgap gradients and a transverse piezoelectric field. These results might facilitate new approaches for energy conversion and sensing applications via strained nanowires and fibers.

Semiconductor nanostructures have been widely utilized in electronic, optoelectronic, photonic and piezotronic devices, such as light-emitting diodes, lasers, photodetectors, flexible displays and sensors^1^,^2^,^3^. Notable advantages are that these nanomaterials can withstand ultra-large elastic strain and exhibit high strength^4^,^5^,^6^,^7^. Thus, strain engineering can be a feasible approach to discover new physical and chemical properties of nanomaterials and to explore new fabrication methods and functions of these nanodevices^8^,^9^,^10^,^11^,^12^. For instance, a tensile strain could be applied to modify the energy band structure and enhance the radiative recombination to improve light emission^13^. The piezo-phototronic effect has been applied in LEDs through a compressed ZnO wire array^14^,^15^,^16^. The inhomogeneous strain of atomic monolayers and nanowires can effectively concentrate the excitons and charged carriers^17^,^18^,^19^,^20^.

Wurtzite ZnO is a direct, wide-bandgap semiconductor with a large exciton binding energy (60 meV). Stimulated excitonic emission in ZnO nanowires and microwires can be observed even at room temperature^21^. Because elastic strain can tune the lattice spacing and thus the electronic structures, large strain gradients have significant effects on the optical properties of ZnO wires^22^,^23^,^24^,^25^. Luminescence properties of ZnO wires under axial tensile and bending strains have been reported by several groups^26^,^27^,^28^. The initial study of the redshift and broadening of near-band-edge (NBE) emission of bent ZnO nanowires was reported by Han *et al*. in 2009^22^. Yan *et al*. reported that the intensity variation of the phonon replica could attributed to the phonon-exciton interaction by bending effects^24^. Subsequently, several studies reported the effects of different deformation potentials on the NBE band shift of ZnO nanowires and microwires^29^,^30^,^31^,^32^,^33^. Recently, it was found that the neutral-donor-bound excitons of bent ZnO microwires drifted towards and emitted photons at the tensile side through a hopping process at liquid helium temperature^34^,^35^,^36^. In addition, it was reported that a transverse piezoelectric field could produce separated electrons and holes that drifted to the tensile and compressive sides, respectively, which led to a net redshift of free-exciton PL emission in the bent ZnO nanowire^37^. Strain and strain gradients can significantly tune the exciton emission in ZnO nanowires and fibers.

In this work, we developed an *in situ* deformation-cathodoluminescence (CL) measurement approach. The strong enhancement of free-exciton related emission was revealed, in particular, a free-exciton interaction that induced a prominent emission (FXI band) under large strain gradients was uncovered. This resulted in a redshift of the whole NBE emission. We proposed the underlying mechanisms of the evolution of excitonic emission based on a large bandgap gradient and a transverse piezoelectric field in the bent ZnO fibers.

## Results and Discussion

### Temperature dependent exciton emission of strain-free ZnO Fiber

**Figure 1:**
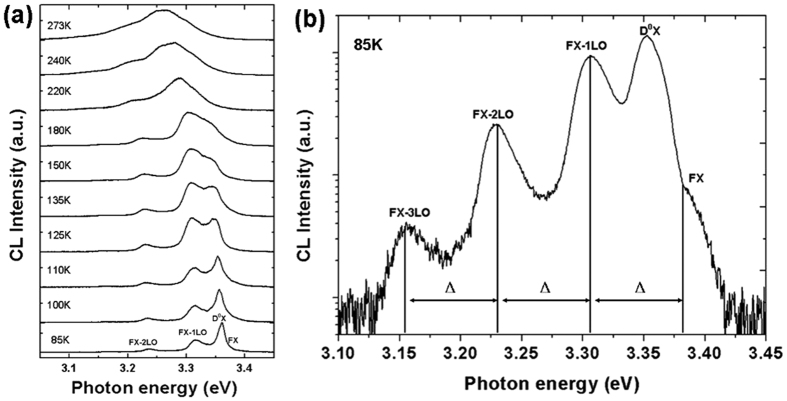
CL spectra of a strain-free ZnO fiber changed with temperature. (**a**) A series of CL spectra with temperatures between 85 and 273 K. (**b**) An enlarged CL spectrum at 85 K. The vertical scale is logarithmic, and the energy space Δ is 72 meV.

A series of CL spectra of a strain-free ZnO fiber at different temperatures between 85 and 293 K for comparison with those of fibers under tensile and bending strains (Fig. 1) were measured. At liquid nitrogen (LN) temperature, five visible peaks were acquired, including the free-exciton peak (FX_A_), the neutral-donor-bound exciton peak (D^0^X), and the first-, second-, and third-order longitudinal optical phonon replicas of the free-exciton peak (FX_A_-1LO, FX_A_-2LO, and FX_A_-3LO). However, only one widened band was acquired above 273 K, which overlapped the FX_A_ and FX_A_-nLO peaks.

Note that the FX_A_ peak was weak as a shoulder of the D^0^X peak at 85 K. The FX_A_-1LO peak first increased with rising temperature; then, it merged into a widely dominating band owing to the increased interaction between phonons^25^. The D^0^X peak was a dominating emission at 85 K, then it weakened and finally faded with the temperature increasing to ∼180 K owing to thermal ionization^38^,^39^. The results indicated that the bound excitons were easily dissociated by thermal ionization because the bound-exciton (BX) dissociation energy (*E*_BX_), which was localized at the neutral donor positions, was below the free-exciton (FX) dissociation energy (*E*_FX_)^40^,^41^. Furthermore, the energy space Δ (roughly 72 meV) indexed an evenly spaced energy among the FX_A_, FX_A_-1LO, FX_A_-2LO, and FX_A_-3LO peaks^21^. This facilitated the recognition of peak positions in the strained cases.

### Evolution of exciton emission of total cross-section in tensile-bending processes

**Figure 2:**
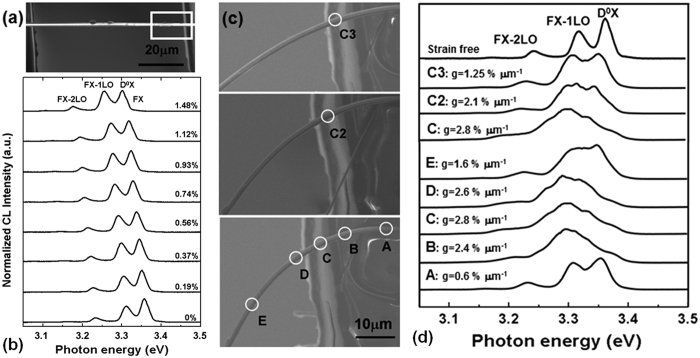
A series of CL spectra of a 1.6-μm ZnO fiber in a tensile-bending process at 85 K. (**a**) A SE image of the fiber under tensile strain. The white square is the position of the bending loading after the fiber had broken. (**b**) CL spectra of the fiber under tensile strains of *ε*_cT_ = 0∼1.48%. (**c**) Three SE images of the bent fiber with different curvatures. The white circles are the collection sites of the CL spectra. (**d**) CL spectra of the same sites on the bent fiber under strain gradients of *g* = 2.8, 2.1, and 1.25% μm^−1^ (matched to the sites of C, C2, C3 in **a**,**b**), and CL spectra of different sites on the bent fiber under different strain gradients of *g* = 0.6∼2.8% μm^−1^ (matched to the sites of A-E in **b**).

We adopted an *in situ* deformation-CL measurement system to collect CL spectra from the total cross-section of a ZnO fiber. A high energy of 15∼30 keV was used to obtain a strong spectral intensity. Figure 2 shows the CL spectra of a high-quality ZnO fiber with a diameter of 1.6 μm under tensile and bending strains at 85 K. Figure 2a and c show the secondary electron (SE) images of the ZnO fiber in the axial tensile and bending states, respectively. Figure 2b and d show the corresponding CL spectra of the tensile strain (*ε*_cT_) and bending strain (*ε*_cB_) conditions, respectively. Figure 2d shows the spectra collected from the same site on the bent fiber with different curvatures (labeled C, C2 and C3), and the spectra collected from different sites of the bent fiber (labeled A-E). The strain measurements and calculations, including the axial tensile stress and strain (*σ*_cT_ and *ε*_cT_) as well as the bending strain and strain gradient (*ε*_cB_ and *g*) are explained in the Supplementary Information, Figure S3.

With increasing tensile strain (*ε*_cT_ = 0∼1.48%), the D^0^X, FX_A_ and FX_A_-1LO peaks linearly redshifted due to the homogeneously decreased bandgap (Fig. 2b). With increasing strain gradient to *g* = 0∼2.8% μm^−1^, the total NBE band nonlinearly redshifted (Fig. 2d,e). Under the higher strain gradients from *g* = 1.6 to 2.8% μm^−1^, the D^0^X and FX_A_-1LO peaks broadened, overlapped and merged into a wide band, while the FX_A_ and its phonon replicas (LO) became difficult to resolve in the spectra. These observations agree with the previous reports^24^,^32^,^37^.

### Evolution of cross-sectionally resolved exciton emission in tensile-bending processes

To further clarify the evolution of the excitonic emission in the bending strain process, especially under the high strain gradients, we acquired cross-sectionally resolved CL spectra from the tensile edge to the compressive edge of the ZnO fiber through a point-by-point linescan across the radial direction of the fiber. A beam energy of 5∼10 keV was used to obtain a high spectral resolution. More than 20 spectra along a whole cross-section of a 1.6-μm ZnO fiber were obtained (Figure S3).

**Figure 3:**
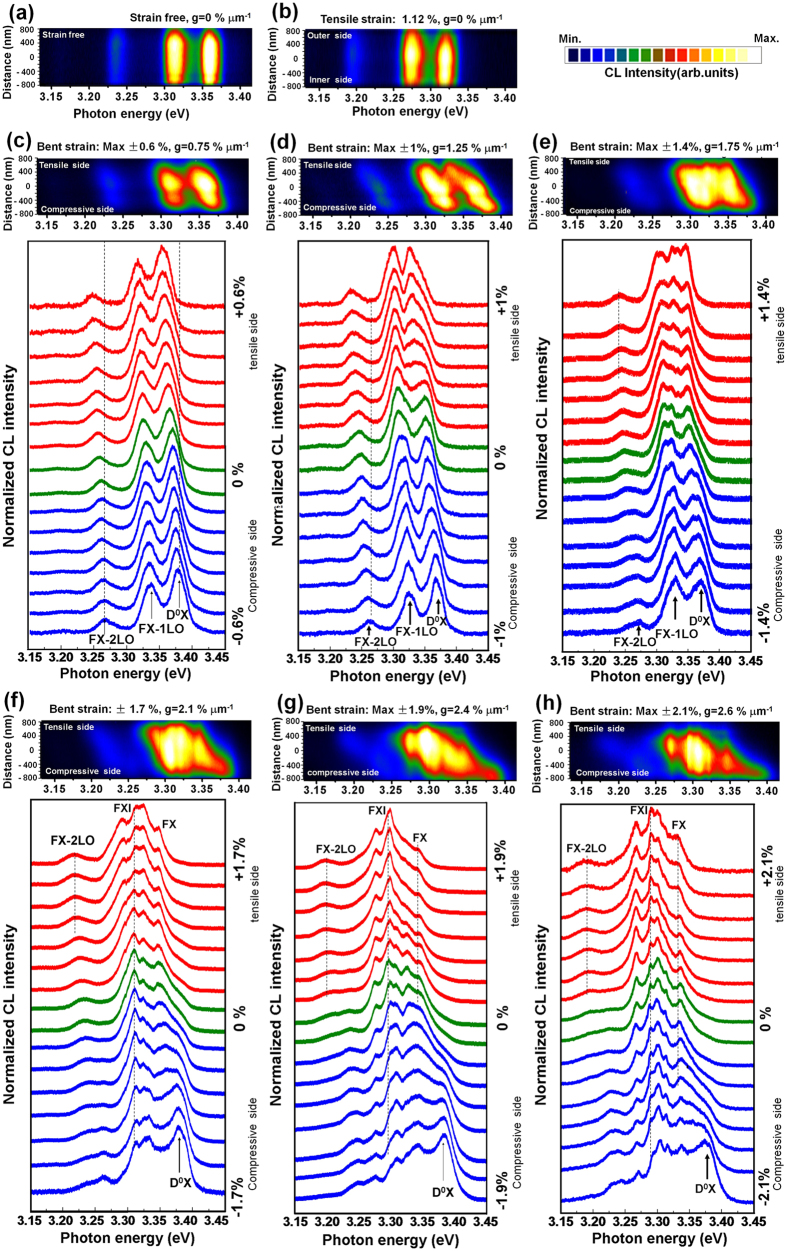
A series of energy-intensity distribution maps (top) and cross-sectional CL spectra (bottom) of the 1.6-μm ZnO fiber under different strain conditions. (**a**) CL map of the strain-free condition. (**b**) CL map of the tensile condition *ε*_cT_ = 1.12%. (**c**,**d**) CL map and a series of spectra of the bent ZnO wire under strain gradients of 0.75 and 1.25% μm^−1^; the phonon replicas display a symmetric energy shift across the section. (**e**–**h**) The maps and matched CL spectra of the bending condition with increased strain gradients of *g* = 1.75, 2.1, 2.4, and 2.6% μm^−1^. The NBE band shows an asymmetric shift along the cross-section. (The right y-axis is the distance of the cross-section between the compressive and tensile edges vertical to the c-axis of the fiber. The vertical dashed lines in the spectra are a guide for the eye).

Figure 3 shows the cross-sectional energy-intensity distribution maps (top) and the corresponding CL spectra (bottom) in the strain-free (Fig. 3a), tensile (Fig. 3b) and bending (Fig. 3c–h) strain conditions. Compared with the spectra obtained from the tensile and low bending strain conditions, some unusual phenomena were observed in the spectra obtained from the high bending strain condition.

In the tensile process with increased strain up to *ε*_cT_ = 1.12%, the D^0^X, FX_A_-1LO and FX_A_-2LO peaks exhibited a linear redshift across the total cross-section of the fiber (Fig. 3b). It indicates that homogeneous strain uniformly changes the bandgap, which symmetrically alter the shift of exciton emissions along the cross section^42^.

**Figure 4:**
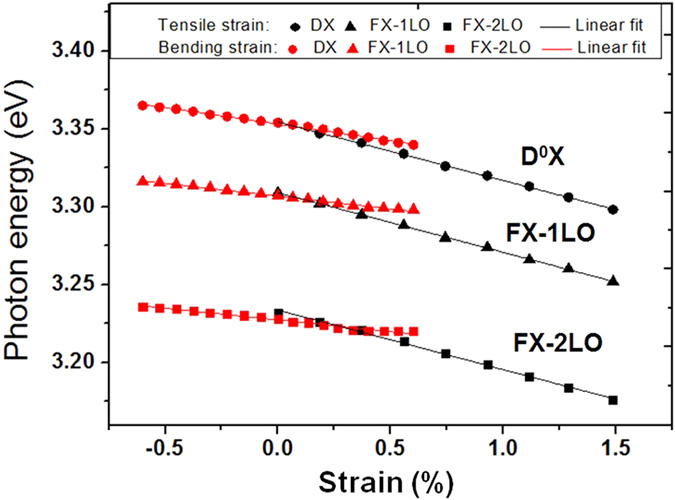
The strains versus the photon energy of the D^0^X, FX_A_-1LO, and FX_A_-2LO of the ZnO fiber (*D* = 1.6 μm). The tensile strain ranged from *ε*_cT_ = 0∼1.48% (black), and the low bending strain ranged from *ε*_cB_ = −0.6∼+0.6% (red).

For the bending process of ZnO fiber under low strain gradient (*g* = 0.75% μm^−1^), the D^0^X, FX_A_-1LO and FX_A_-2LO peaks exhibited a symmetric variation in energy and intensity along the cross-section (Fig. 3c). Figure 4 shows a comparison of energetic variations of the photonic emission and the strains in the axial tensile and low bending strain conditions. A linear shift of the D^0^X, FX_A_-1LO and FX_A_-2LO peaks can be observed under both tensile (*ε*_cT_ = 1.48%, black line) and low bending (*ε*_cB_ = ±0.6%, *g* = 0.75 μm^−1^, red line) strains^42^. This means that the energy variations under the low strain gradient could be mainly attributed to the deformation potential (∂*E*/∂*ε*_c_), i.e., the response of the energy bandgap to the strain^43^,^44^. However, the absolute values of the ∂*E*/∂*ε*_c_ under the low bending strain (∂*E*_DX_/∂*ε*_cB_ = ±21 meV/% for *ε*_cB_ = ±0.6%) were smaller than those under the tensile strain (∂*E*_DX_/∂*ε*_cT_ = −38 meV/% for *ε*_cT_ = 0.6%).

Under high strain gradient (*g* ≥ 2.1% μm^−1^), the total NBE band exhibited an asymmetric energy-intensity variation along the radial direction of the fiber relative to the cross-sectional center as a reference plane (Fig. 3f–h). Furthermore, the D^0^X peak was suppressed and faded, while the FX_A_ peak was obviously increased at the tensile side. In particular, a new emission emerged at the tensile side, which became a dominating emission in the spectra.

### The evolution of the total NBE band

The total NBE band, roughly including the FX_A_, D^0^X and FX_A_-1LO peaks in the ZnO fiber under large strain gradient, significantly broadened and exhibited an asymmetric energy-intensity distribution along the radial direction (Fig. 3f–h). At the tensile side (*ε*_cB_ > 0), the energy distribution of the band ranged from 61 to 72 meV, while, at the compressive side (*ε*_cB_ < 0), the energy distribution of the band ranged from 65 to 105 meV, as shown in Fig. 3e and h.

### The evolution of the free-exciton (FX) emission and FX phonon replicas

**Figure 5:**
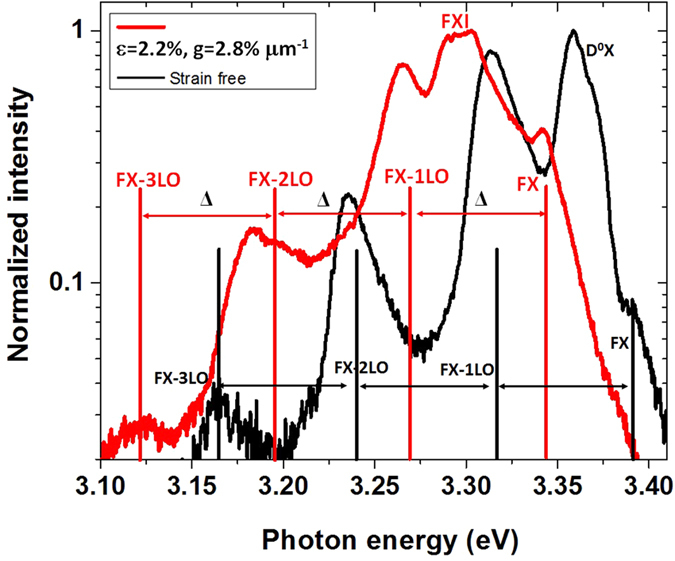
The logarithmic spectra of a ZnO fiber (*D* = 1.6 μm) in the strain-free (black) condition and at the tensile edge in the bending strain condition (*g* = 2.6% μm^−1^). The energy space was Δ = 72 meV for the strain-free and the bending strain conditions.

Under the large strain gradients of *g* = 2.4∼2.6% μm^−1^ (Fig. 3f–h), the redshifted FX_A_ peak is obviously observed along the cross section and FX_A_ peak stays at a constant energy. This suggested that free excitons aggregated and recombined to emit photons at narrow bandgap of the tensile edge under the bandgap gradient^34^,^45^. Furthermore, the redshift of the FX_A_ and FX_A_-nLO peaks could be confirmed by the equal energy space (Δ) between the FX_A_ and LO phonon peaks, as the Δ (71–73 meV) corresponds to the distances between the FX_A_ and FX_A_-1LO peaks in both the strain-free and bending strain conditions (black and red lines in Fig. 5). In the large bending strain case, the FX_A_ peak became visible, the FX_A_-1LO and FX_A_-2LO peaks were easy to recognize. As shown in Fig. 3e–h, FX_A_-2LO peak became two lines, and the energy range between the two lines gradually increases with increasing strain gradient.

In addition, a new emission emerged and became a prominent emission band at both tensile and compressive sides when the strain gradient *g* ≥ 2.1% μm^−1^ (Figs 3f–h and 5). We named it the FXI band because it was induced by an exciton-exciton interaction process among aggregated free excitons in the narrow bandgap. In principle, the inelastic interaction between two free excitons in their ground state leads to the scattering of one free exciton into a photon-like state, and the emission of photons, while the other free exciton is scattered into an excited state^46^,^47^,^48^. Figure 5 shows that the FXI band was roughly located between 3.286 and 3.302 eV. The obviously enhanced FX_A_ peak and prominent FXI band with constant energy along the cross section revealed that the migrated free excitons recombine at narrow bandgap.

**Figure 6:**
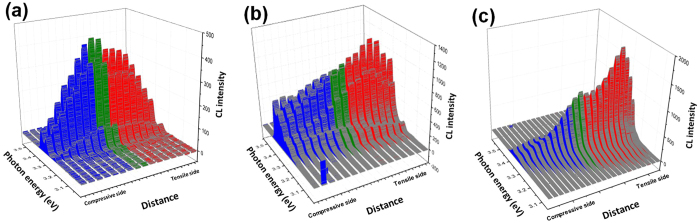
The energy-intensity distributions of the cross-sectional NBE emission of the bent ZnO fiber. (**a**) *D* = 1.6 μm, *g* = 0.75% μm^−1^, (**b**) *D* = 1.6 μm, *g* = 2.6% μm^−1^, and (**c**) *D* = 1.2 μm, *g* = 3.5% μm^−1^.

The FXI band contributed an intensified intensity of the photon emission and prompted the total NBE band redshift under the large strain gradients. Figure 6 shows the energy-intensity distribution of the cross-sectional NBE emission, which was related to the strain levels of the ZnO fiber. Under the small strain gradient of *g* = 0.75% μm^−1^ for the 1.6-μm fiber (Fig. 6a), the intensity of the total NBE band exhibited a normal distribution along the cross-section due to the gradually reduced interaction volume and excitation intensity from the center to both sides under e-beam irradiation. It indicates that there is no diffusion of excitons at this strain gradient. Under the increased strain gradient of *g* = 2.6% μm^−1^ (*D* = 1.6 μm), the generated free excitons at the incident e-beam position can move towards the tensile edge within their lifetime and emit photons at this narrow bandgap, only a portion of excitons recombine at the e-beam position. When the excitation position of e-beam locates at the tensile edge, all of the excited excitons stay at the narrow bandgap and emit photons. Therefore, the intensity distribution decreased from tensile edge to compressive edge. The intensity of the NBE band at the tensile edge increased by 2-fold compared with that at the compressive edge (Fig. 6b). Under the increased strain gradient of *g* = 3.5% μm^−1^ (*D* = 1.2 μm), the intensity at the tensile edge increased by 12-fold (Fig. 6c and Figure S4). Furthermore, the cross-sectional NBE band of the bent fiber exhibited a larger redshift of approximately 50 meV when *g* = 3.1% μm^−1^. The obviously enhanced FX_A_ peak and FXI band along the cross section support the capability of the drifts of free excitons in the presence of a large bandgap gradient. The drift, aggregation, and emission of the free excitons at the tensile side result in the asymmetric energy-intensity distribution along the cross-section.

### The evolution of the bound-exciton emission

The D^0^X peak was visible along the cross section under the low strain gradient of *g* = 0.75% μm^−1^, which suggested that most bound excitons were not ionized and emitted at the e-beam excited sites. The D^0^X peak showed a linear shift at the compressive side, while it showed a nonlinear shift at the tensile side when *g* = 1.25% μm^−1^ (Figure S5). With increasing strain gradient, the D^0^X peak was gradually suppressed and finally faded from the compressive edge to the tensile edge with increasing strain gradient (Fig. 3e–h). Under the higher strain gradients, *g* ≥ 2.6% μm^−1^, the D^0^X peak disappeared at the tensile side. This indicated that the bound excitons were gradually impact ionized with the aid of piezoelectric field. According to the bending strain-piezoelectric field relationship and experimental reports^49^, when the strain gradients is larger than 2% μm^−1^, the transverse built-in electric field (E_P_) is still less than the electric field of 5 × 10^4^ V cm^−1^ for direct field ionization of bound exciton^50^,^51^. Although electric field can’t reach the threshold for field ionization, the impact ionization would occur among excited bound excitons and aggregated electrons.

**Figure 7:**
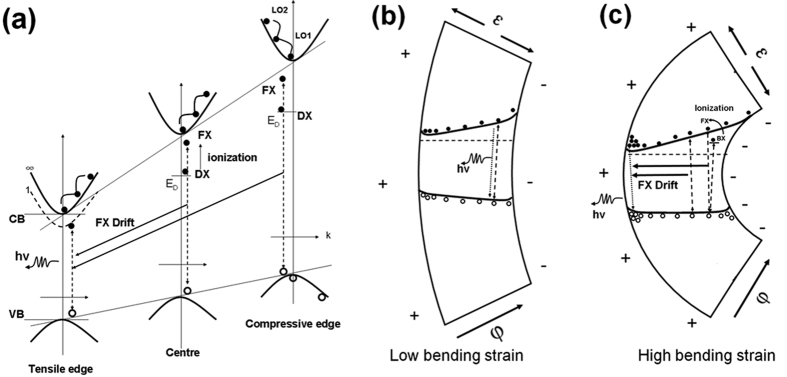
A schematic of a gradient bandgap structure of a ZnO fiber. The strain gradient creates a continuously varying bandgap and a transverse piezoelectric field. (**a**) Under a large bandgap gradient, free excitons (FX) drift towards the tensile side and emit photons via a radiative recombination in the narrow bandgap, while bound excitons (BX) are ionized at irradiation sites of the e-beam by the piezoelectric field. (**b**) Low bending strain induces a small bandgap gradient and a low piezoelectric field. The FX and BX recombine at the excited site. (**c**) High bending strain induces a large bandgap gradient and a high piezoelectric field, in which more free excitons aggregate and recombine in the narrow bandgap, and BX convert into FX.

**Figure 8:**
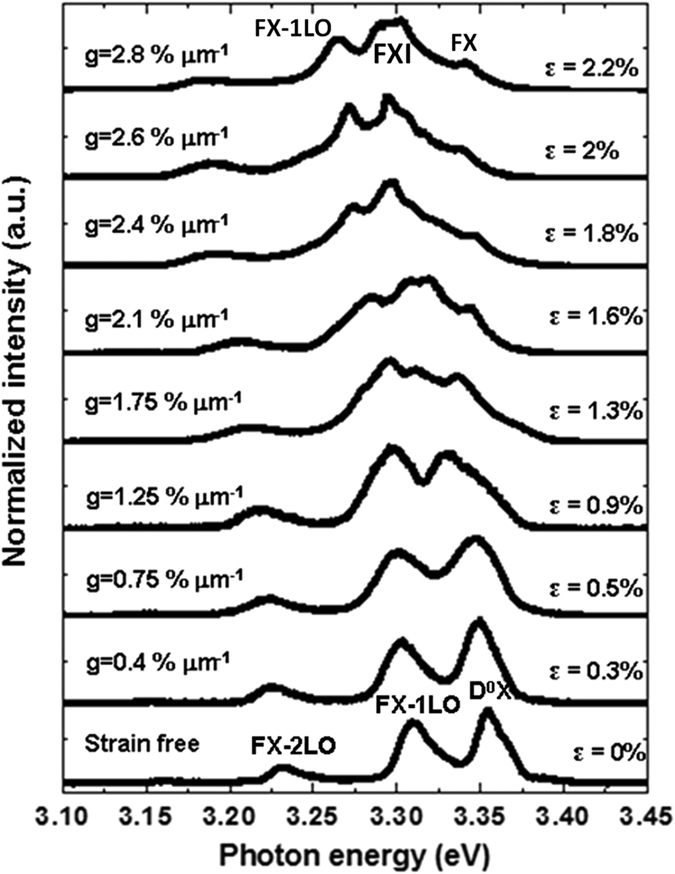
The evolution of FX, D^0^X and FXI at the tensile edge of the 1.6-μm ZnO fiber under different strain gradients (*g* = 0∼2.8% μm^−1^).

To further understand the evolution of free-exciton and bound-exciton emission under the high bending strains, we proposed a combination mechanism based on the effects of a gradient bandgap structure and a transverse, built-in piezoelectric field, as illustrated in Figs 7 and 8.

Strain gradient results in a continuous bandgap variation, this energy gradient is the driving force for exciton migration. Under small strain gradients, *g *< 1.25% μm^−1^ (Figs 3c,d and 7b), the bandgap gradient is less than 26 meV/μm, which is not sufficient high to drive the exciton. The free excitons (FX) and bound excitons (DX) in uniformly varied bandgap emit photons at the e-beam irradiated sites (Fig. 7b). The major contribution to the shifts of excitonic emission comes from the bandgap deformation potential, which is similar to that in the axial tensile strain (Fig. 4). However, under high strain gradients (*g* > 2.1% μm^−1^, Figs 3f–h and 7c), the bandgap gradient became larger than 44 meV/μm, the movement of excitons became possible. The evolution of the free excitons and bound excitons are quite different. First, the free excitons excited by the incident e-beam drifted towards and concentrated at the narrow bandgap within their lifetime in the presence of a large bandgap gradient^34^,^52^. As a result, the recombination of drifted free excitons and free exciton interaction emission formed an asymmetric energy-intensity distribution along the cross-section and a nonlinear redshift of the total NBE band of the ZnO fiber. Second, the bound excitons (DX), which were bound in the donor defect sites, would rather dissociate under the piezoelectric field than move by hopping process from one donor to another under the bandgap gradient. They were converted into free excitons (FX) and neutral-donor-like defect-pair complexes (*D*) i.e., *DX* → *FX* + *D*^50^,^51^. Moreover, the movement of excitons became possible even at LN temperature. The exciton mobility would decrease due to the larger phonon and defect scattering rates, the thermally activated non-radiative recombination could shorten the drift length of excitons due to the shorter exciton lifetime^35^. Therefore, we may only observe the drift of free exciton through brownian motion in ZnO fiber with high strain gradient. The hopping motion of bound excitons could be ignored at LN temperature because the bound excitons can only hop to move at low temperatures below 25 K, and the hopping speed dramatically decreases with temperature increases due to thermally activated backward motion of the excitons^53^.

Furthermore, the effect of the transverse, built-in piezoelectric field on the bandgap variation could be estimated by a bending strain-piezoelectric field relationship^49^,^54^,^55^. For instance, a piezoelectric field larger than 10^4^ V cm^−1^ (*ε*_cB_ > 1.0%) in the ZnO wire could result in a bandgap shift of ∼10^−1^ meV^56^. This means that the effect of the piezoelectric field on the variation of bandgap could be ignored. Based on our observations, the bound excitons were predominantly dissociated with the aid of piezoelectric field and then increased the concentration of free excitons. Therefore, a high-density exciton region formed at the tensile edge. The interaction and recombination of these free excitons resulted in a prominent FXI emission through an inelastic scattering process in the narrow bandgap. In this sense, the piezoelectric field could be a contributor to the shift of the total NBE band as well.

In summary, we investigated the evolution of the excitonic emission of individual ZnO fibers in tensile-bending strain processes using spatially resolved CL spectrometry combined with an *in situ* deformation approach at LN temperature. Under low bending strains, the FX_A_, FX_A_-nLO and D^0^X peaks exhibited a linear redshift, similar to that observed under tensile strain, which was mainly attributed to the bandgap deformation potential. Under high bending strains (*g* > 2.1% μm^−1^), the free excitons drifted towards and aggregated at the tensile side. This resulted in an enhanced free-exciton emission in the narrow bandgap, and in particular, an emergence of a prominent emission in the narrow bandgap, which was generated from a strong free-exciton interaction (FXI). Meanwhile, the bound excitons were dissociated into free excitons and neutral donors under the transverse piezoelectric field, which also contributed to the enhancement of the free-exciton-related emission. Consequently, the exciton emission showed an asymmetric energy-intensity distribution along the cross-section of the fiber, and the total NBE band showed a nonlinear redshift. The inhomogeneous strain-modulated drift, aggregation, and dissociation of the excitons in the polar ZnO could be used for designing and improving piezo-phototronic and optical detection devices.

## Experimental Section

### Synthesis of the ZnO fibers

The ZnO fibers were fabricated on a Si substrate by the thermal evaporation with a high crystalline quality (Supplementary Information, Figure S1)^57^.

### Strain measurements of the ZnO fibers

During the axial tensile-bending process, a piezo-manipulator (Kleindienk^TM^) was used to perform elongating and bending measurements (Figure S2). The ZnO fiber was first elongated until broken, and then was bended *in-situ*. For accurately loading on the ZnO fiber, two ends of a fiber were fixed by the epoxy glue on two silicon cantilevers. The measurements and calculations for the tensile stress (*σ*_cT_) and strain (*ε*_cT_)^42^, as well as for the bending strain (*ε*_cB_) and strain gradient (*g*) of the individual ZnO fibers are explained in Supplementary Information (Equation S1–S4, and Figure S3).

### CL measurements of the ZnO fibers

An *in situ* deformation-cathodoluminescence (CL) measurement system is built in a field-emission environmental scanning electron microscope (ESEM), combined a home-made tensile-bending setup and a spatially-resolved CL spectroscope with a liquid nitrogen cooling stage (Figure S2). The ESEM (FEI Quanta 600 F) has a resolution of 1.2 nm, and the CL spectroscope (GATANMONO3PLUS) has a precision of ~0.66 nm. The measurement conditions include: an accelerating voltage of 5~30 kV, a beam current of 10^−8^~10^−10^ A, a working distance of ~12.6 mm, and a PMT detector with a grating of 1200 l/mm for collecting CL spectra with a high precision. For collecting the whole cross-sectionally spectra, the beam energy of 15∼30 keV was used to obtain a strongly spectral intensity. For collecting the cross-section resolved spectra across the radial direction from the outer tensile edge to the inner bending edge of the fiber, the beam energy of 5∼10 keV was used to obtain a highly spectral resolution via a point-by-point linescan irradiation of the e-beam. The scan step was less than ∼50 nm for the ZnO.

## Additional Information

**How to cite this article**: Wei, B. *et al*. Strain Gradient Modulated Exciton Evolution and Emission in ZnO Fibers. *Sci. Rep.* **7**, 40658; doi: 10.1038/srep40658 (2017).

**Publisher's note:** Springer Nature remains neutral with regard to jurisdictional claims in published maps and institutional affiliations.

## Supplementary Material

Supplementary Information
