# Supplementary material for: Strain Gradient Modulated Exciton Evolution and Emission in ZnO Fibers
Source: Sci Rep. 2017 Jan 13;7:40658. doi: 10.1038/srep40658 (PMC5234005; doi:10.1038/srep40658)
Supplement: Supplementary Information [file srep40658-s1.doc]

Supplementary Information

**Strain Gradient Modulated Exciton Evolution and Emission in ZnO Fibers**

Bin Wei^1, 2^, Yuan Ji^1,*^, Raynald Gauvin^2^, Ze Zhang^3^, Jin Zou^4^ & Xiaodong Han^1,*^

^1^ Beijing Key Laboratory and Institute of Microstructure and Property of Advanced Materials, Beijing University of Technology, Beijing 100124, China.

^2^ Materials Engineering, McGill University, Montréal, Québec, H3A 0C5, Canada.

^3^ Department of Materials Science, Zhejiang University, Hangzhou 310027, China.

^4^ Materials Engineering and Centre for Microscopy and Microanalysis, The University of Queensland, St. Lucia, QLD 4072, Australia.

Correspondence and requests for materials should be addressed to Y.J. (email: jiyuan@bjut.edu.cn) or X.D.H. (email: xdhan@bjut.edu.cn)


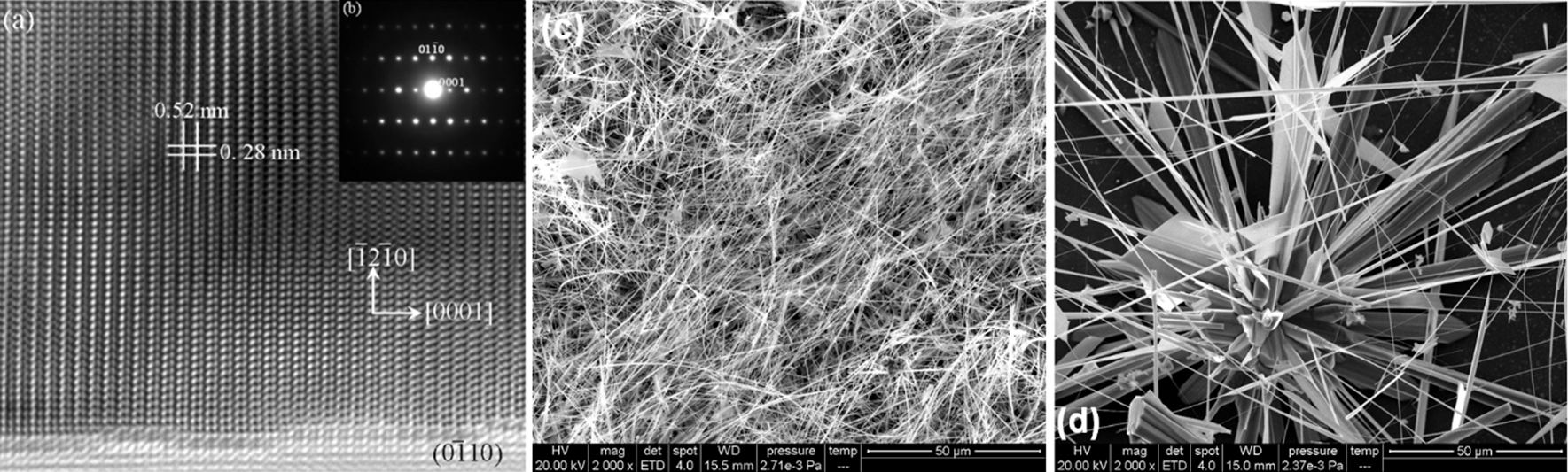


**Figure S1** The [0001]-orientated ZnO fibers characterized by the transmission electron microscope (JEOL 2010F) and SEM (FEI Quanta 600F ESEM). (a) A high-resolution TEM image, and (b) A matched selected-area electron diffraction (SAED). (c, d) The secondary electron images of ZnO nanowires and fibers,


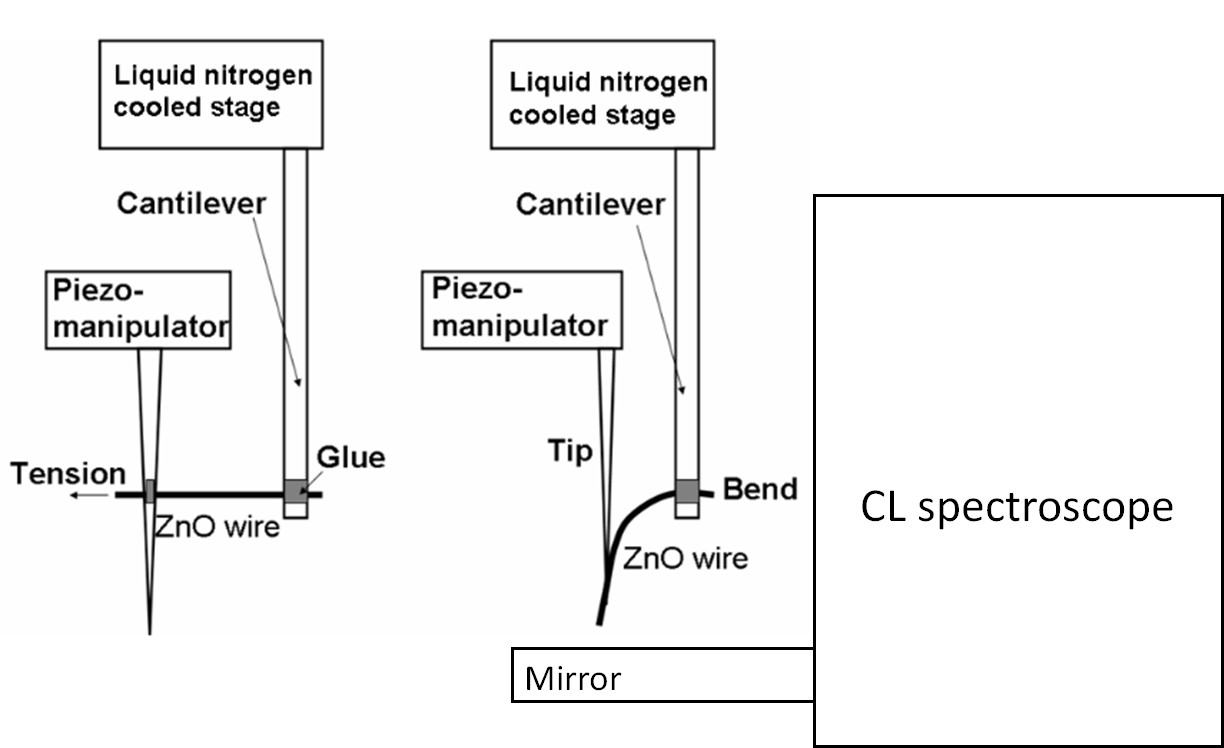


**Figure S2** A schematic of a strain-CL measurement system built in an ESEM, including a tensile-bending strain setup, a CL spectroscope, and a liquid nitrogen cooled stage. The home-made tensile-bending setup includes a piezo-manipulator and a silicon cantilever served to apply the tensile stress and the bending strain after tensile fracture.


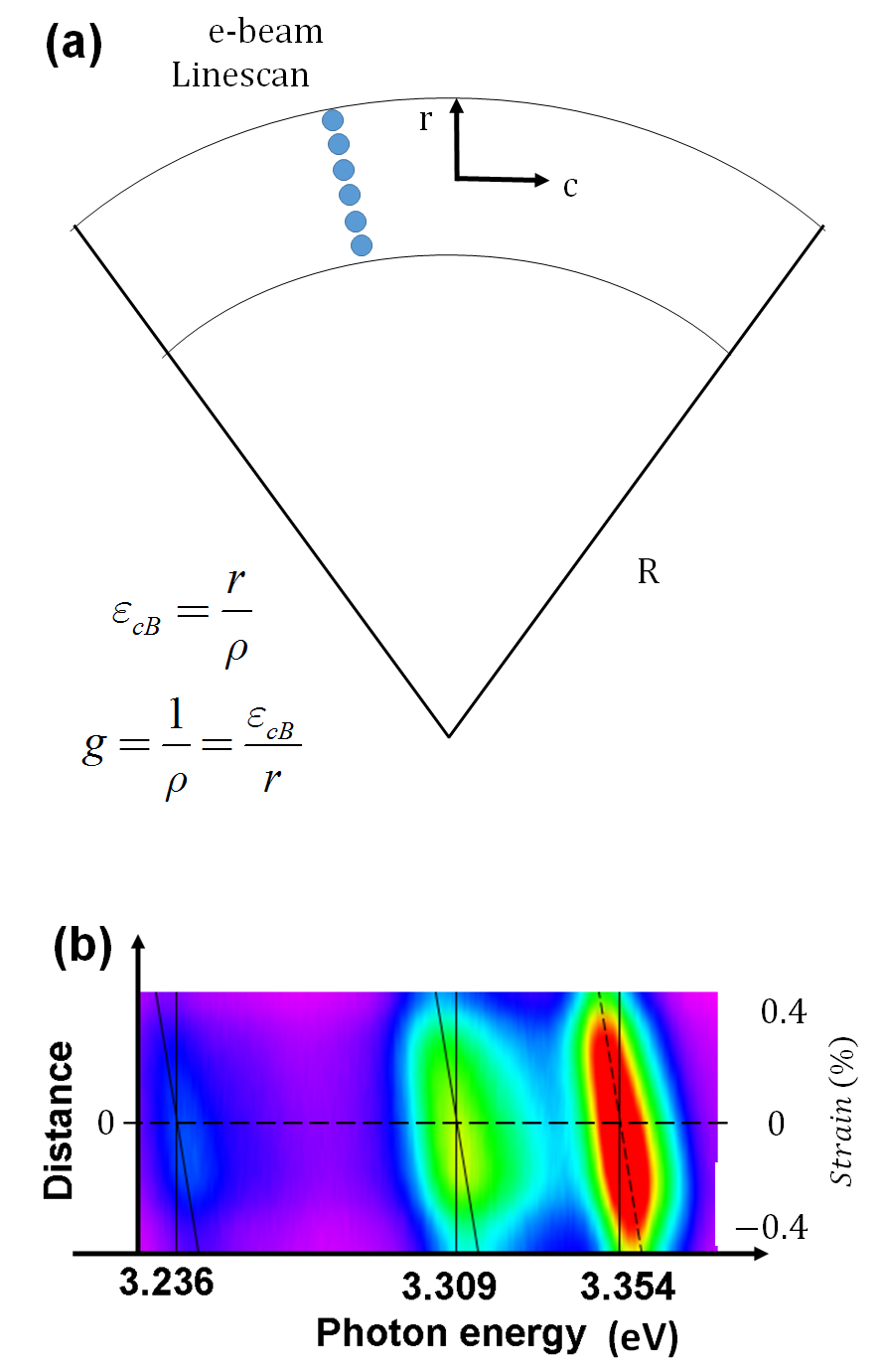


**Figure S3** An example of bending strain measurement and cross-section resolved CL spectra acquisition across the radial direction of ZnO fiber. (a) A schematic of the e-beam irradiation position through a spot-by-spot linescan across the whole cross-section of the ZnO fiber from the outer tensile to the inner compressive edges. (b) A corresponding intensity-energy distribution map of a cross-sectional CL spectrum (*g* = 0.5% μm^-1^).

The strain measurements and calculations of the c-axis tensile stress (*σ*_cT_) and strain (*ε*_cT_) are explained by Eqs. S1-S2.

$\varepsilon_{cT}=\frac{\sigma_{cT}}{E_{3}}=\frac{1}{E_{3}}\frac{K\delta}{S}$ (S1)

$\sigma_{cT}\text{ }=\text{ 4}K\delta/\pi D^{2}$ (S2)

where *D* is the diameter of ZnO Fibers, $K$is a force constant of the Si cantilever, $\delta$is a displacement of the Si cantilever, Young’s modulus in [0001]-direction (for the bulk ZnO, *E_3_* = 140 GPa), *σ_cT_* is the axial stress on the ZnO fiber, *S* is the area of cross-section of the fiber. The loading force was determined by a deflection magnitude of the Si cantilever. According to Eq.S1, the strain applied on the ZnO fiber can be determined by the change of *δ*. The *δ* was measured by a deflection of the Si cantilever from the SE image.

The bending strain (*ε*_cB_) and strain gradient (g) were calculated by Eqs.S3-S4 and Figure S1.

$\varepsilon_{cB}=\pm\frac{r}{R}$ (S3)

$g=\frac{\varepsilon_{cB}}{r}=\frac{1}{\rho}$ (S4)

where the *ε*_cB_ is the c-axis bending strain, *r* is the radius of the ZnO fiber,$\rho$is the curvature radius of the bent fiber, $\rho$is measured from the SE image of the bent ZnO fiber.


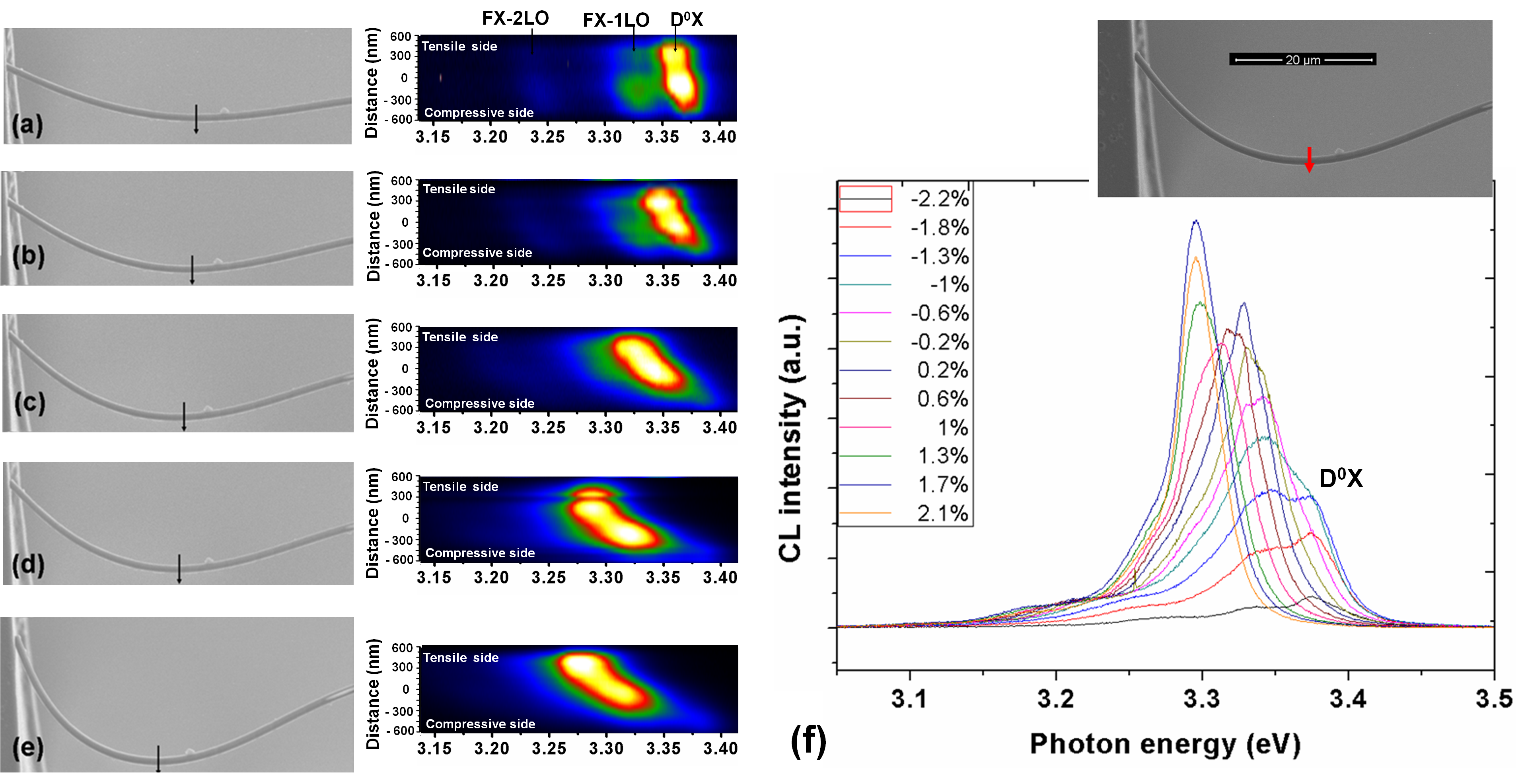


**Figure S4** (a-e) SE images and the corresponding CL maps of 1.2 μm-ZnO fiber under the strain gradients from 0.6 to 3.5% μm^-1^ at 85 K. (f) The cross-sectional resolved NBE band under the strain gradients of g = 3.1% μm^-1^. The band at 3.28-3.30 eV contain FXI emission.

Figure S4 shows the CL data of another 1.2 μm-ZnO wire by *in-situ* tension-bending process. With the increasing strain gradient of bent wire, the asymmetric red-shift becomes obvious. D^0^X peak shifted from high energy to low energy under low strain gradient, but the D^0^X gradually suppressed and the whole band showed a larger red-shift with the increasing strain gradient. Especially, the whole band at cross-sectional neutral plane showed a larger red-shift. Besides, the intensity of CL spectra on the tensile side is larger than the compressive side of bent wire


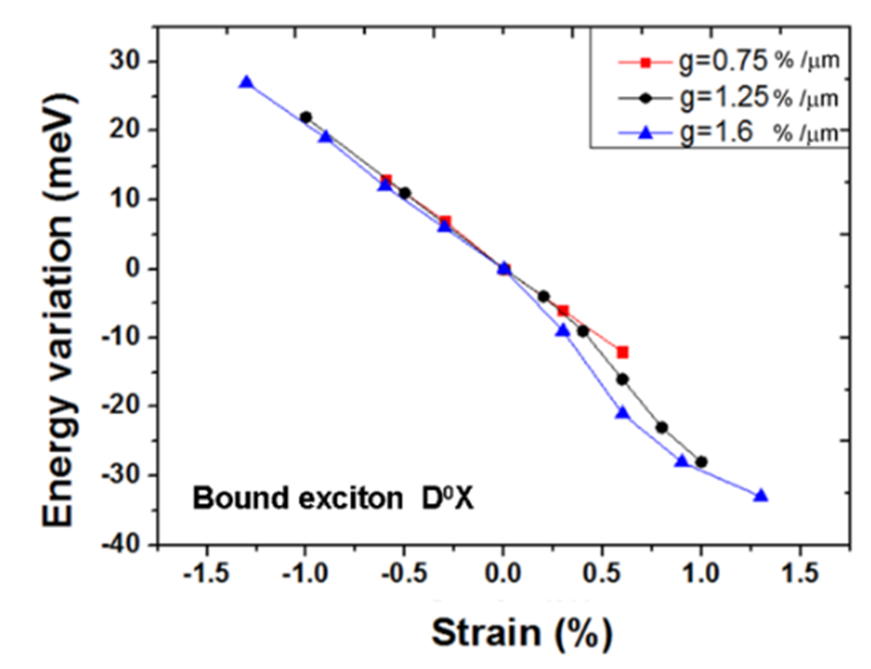


**Figure S5**. The energetic variation of the D^0^X versus bent strain (*g* = 0.75-1.6 % μm^-1^)
